# Spectrum of Primary Gastric Lymphoma in India: A Series of 30 Patients

**DOI:** 10.5152/tjg.2022.21052

**Published:** 2023-02-01

**Authors:** Akash Mathur, Uday C. Ghoshal, Sushil Kumar, Neeraj Kumari, Narendra Krishnani

**Affiliations:** 1 Department of Gastroenterology, Sanjay Gandhi Postgraduate Institute of Medical Sciences, Lucknow, India; 2 Department of Pathology, Sanjay Gandhi Postgraduate Institute of Medical Sciences, Lucknow, India

**Keywords:** Chronic gastritis, dyspepsia, gastric cancer, gastric resection, gastric ulceration, gastrointestinal lymphoma, Helicobacter pylori

## Abstract

**Background:**

Primary gastric lymphoma is uncommonly reported in India. We retrospectively analyzed their data in a northern Indian teaching hospital.

**Methods:**

During a 12-year period (2000-2012), endoscopic and surgical biopsies were assessed for gastric neoplasm. Gastric biopsies from normal-looking areas, rapid urease test, and *Helicobacter pylori* serology were done, with 2 of 3 tests positive being considered diagnostic. We aimed to study (i) the frequency of primary gastric lymphoma among gastric neoplasm patients, (ii) its clinical profile, (iii) the diagnostic procedures needed, and (iv) the frequency of *H. pylori* infection among them.

**Results:**

Thirty out of 324 (9.2%) patients (age 56 years, range 25-72, 73.3% male) with gastric neoplasm had primary gastric lymphoma. Presentations included dyspepsia (n = 9, 30%), gastric outlet obstruction (n = 7, 23.3%), upper gastrointestinal bleeding (n = 5, 16.7%), dysphagia (n = 4, 13.3%), malignant ascites (n = 3, 10%), and others (n = 2, 6.7%). *H. pylori *infection was confirmed in 7 (23.3%), 12 (40%), and 21/29 (72.4%) patients by rapid urease test and histopathology and positive anti-*H. pylori* IgG serology, respectively. By 2 tests, *H. pylori* was detected in 12 (40%) patients. Though in 60% primary gastric lymphoma was diagnosed on endoscopic biopsy, in 40%, surgical resection was required. The endoscopic and surgical diagnosis groups were comparable in age (53.4 years vs. 52.7 years), sex (male 77.8% vs. 66.7%), *H. pylori* infection (38.9% vs. 16.7%), presentation with dyspepsia (38.9% vs. 16.7%), organic symptoms (61.1% vs. 83.3%), and the need for repeated endoscopic biopsies before diagnosis (12.% vs. 33.3%).

**Conclusion:**

Primary gastric lymphoma is not uncommon (9.2%) in India, often missed on endoscopic biopsy and is associated with *H. pylori* infection (40%).

**Main Points**

- These data reveal a high frequency of primary gastric lymphoma (PGL) in the study population (9.2%).
- The study also confirms an association between *Helicobacter pylori* infection (40%) and PGL.
- Repeated negative endoscopic biopsies may delay the diagnosis of PGL; the development of newer diagnostic tools for early detection of PGL is important.

## Introduction

Gastric neoplasms (GN), which include adenocarcinoma and primary gastric lymphoma (PGL), are not uncommon causes of cancer-related mortality worldwide.^1^ However, there is limited data on frequency, clinical spectrum, and outcome of PGL from India. Most of the earlier reports on PGL from India were either case reports or small series.^2,3^ A single study published till date from 2 centers, one from northern, and the other from eastern India showed that 5% of gastric neoplasm in India are constituted by PGL,^4^ a frequency somewhat similar to that in some of the western and developed eastern countries. Moreover, in this study, endoscopic biopsies missed the diagnosis of PGL, and surgically resected specimens often revealed the diagnosis. This finding raises the possibility that PGL might be missed in India unless it is looked for. Moreover, the available data, though scanty, might challenge the myth that PGL is uncommon in India.

As defined by Dawson, PGL involves predominantly the stomach and the lymph nodes corresponding to its lymphatic drainage. Involvement of the other lymph node groups or any extranodal organs (bone marrow, liver, spleen, etc.) in an early stage of the disease practically excludes the diagnosis.^5,6^

*Helicobacter pylori *causing chronic inflammation of the gastric mucosa is an important factor in the pathogenesis of gastric diseases including gastric adenocarcinoma and PGL.^7,8^ However, the data on this issue from India, where *H. pylori* is endemic, are somewhat scanty. A few studies have shown that high *H. pylori* IgG titers are also associated with an increased risk of gastric cancer possibly due to increased expression of a cytotoxin-associated gene (CagA)^9,10^; while the presence of a marked degree of gastric atrophy and intestinal metaplasia (IM) is associated with gastric adenocarcinoma. Primary gastric lymphoma occurs in patients with a lesser degree of gastric atrophy and IM.^11^ Accordingly, we undertook a study with the aim to know (i) the frequency of PGL among patients with gastric neoplasm, (ii) their demographic and clinical profile, (iii) the diagnostic procedures needed, and (iv) frequency of *H. pylori* as compared with a group of historical controls with non-ulcer dyspepsia (NUD, currently called functional dyspepsia).

## MATERIALS AND METHODS

The present study included 30 patients with PGL out of 324 with gastric neoplasms whose data were prospectively maintained on a questionnaire including demographic, clinical, endoscopic, histological, and serological parameters as part of the project initially funded by the Indian Council of Medical Research during a 12-year period (2000-2012) in the department of Gastroenterology at Sanjay Gandhi Postgraduate Institute of Medical Sciences, Lucknow, a tertiary referral center in northern India. As these data were collected as a part of a prospective study initially, the protocol was approved by the Institutional Ethics Committee.

Multiple (6) biopsies were obtained from the tumor margin in 10% neutral buffered formalin during esophagogastroduodenoscopy (EGD) using a video endoscope (Olympus Optical Co Ltd., Tokyo, Japan). To ascertain the type of malignancy, endoscopic biopsy and surgically resected specimens were assessed after hematoxylin and eosin (H & E) staining.

Biopsies from normal-looking areas were also taken to evaluate for *H. pylori,* IM, and gastritis (grading done by updated Sydney system) after H & E and Giemsa staining.^12^ These were assessed by expert pathologists, who were blinded to the endoscopic findings. Rapid urease test (RUT) for *H. pylori* infection was done on one biopsy each from antrum and body. Color change of an in-house solution from yellow to pink over a 2-hour period was used to perform RUT, the same has already been validated in a previous study.^13^ Three microliters of fasting serum sample was taken for serological examination in a plain vial using an enzyme-linked immunosorbent assay for anti-*H. pylori* IgG antibody using a commercial kit (Biohit Oyj, Helsinki, Finland). Antibody concentration was deemed positive at a cut-off value of ≥30 enzyme immune unit. Positive results in any 2 of these 3 tests were considered to be diagnostic of *H. pylori* infection.^5^ Frequency of *H. pylori* infection among patients with PGL was compared with the data of 101 patients with NUD, currently called functional dyspepsia published earlier as controls.^4^

### Statistical Analysis

Continuous variables were presented as mean ± standard deviation or median and range or interquartile range. Categorical variables were analyzed by the Chi-squared (χ^2^) test with Yates correction wherever applicable.

A *P*-value of less than .05 was considered significant. Statistical analysis was performed using R, Epicalc, and R-studio software (R Development Core Team, Vienna, Austria).

## Results

Of 324 patients, 30 (9.2%) had PGL. The median age of patients with PGL was 56 years (range 25-72 years) at diagnosis and 22 patients (73.3%) were male. History of cancer among family members was present in 6 (20%) subjects. Of the 6 patients with a family history of cancers, 1 reported gastric, 2 esophageal 1 colon cancer, and 2 lung cancers among first- and second-degree relatives.

Of 30 patients with PGL, 9 (30%) presented with dyspepsia with or without weight loss, 7 (23.3%) with gastric outlet obstruction (1 each with dyspepsia and gastric outlet obstruction had gastric amyloidosis in addition to the PGL), 5 (16.7%) with upper gastrointestinal bleeding, 4 (13.3%) with dysphagia, 3 (10%) with malignant ascites; in 2 (6.7%), the diagnosis of PGL was established on histopathological examination of resected gastrectomy specimens performed with a suspicion of gastric cancer. One patient with PGL with gastric amyloidosis and the other presenting with acute pancreatitis have been reported previously.^2,14^ More than half of the patients complained of epigastric pain (66.7%), abdominal fullness (55.2%), and indigestion (51.7%).

Most patients (67.9%) reported unintentional weight loss (8.3 ± 3.5 kg weight reduction in 4.3 ± 2.6 months). Alarm symptoms such as anorexia (79.3%) and reduced appetite (65.5%) were often reported; 85.7% had anemia (hemoglobin 9.0 ± 1.6 g/dL).

**Figure 1.**
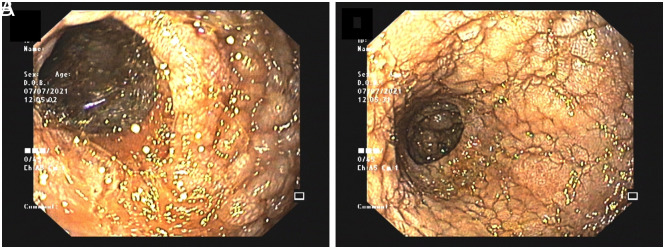
Endoscopy images of a patient with primary gastric lymphoma (PGL) showing nodular gastric mucosa.

Of 28 patients having records of EGD, the lesion was in the antrum of the stomach in 2 (7.1%), body in 9 (32.1%), gastroesophageal (GE) junction in 1 (3.6%), duodenum in 1 (3.6%), and diffuse in 15 (53.6%). The major endoscopic findings were ulceroproliferative growth in 10 (35.7%), ulceration in 7 (25%), nodular growth in 7 (25%), gastric polyp in 1 (3.6%), gastric outlet obstruction in 1 (3.6%), and unclassified in 2 (7.1%) (Figure 1).

**Figure 2.**
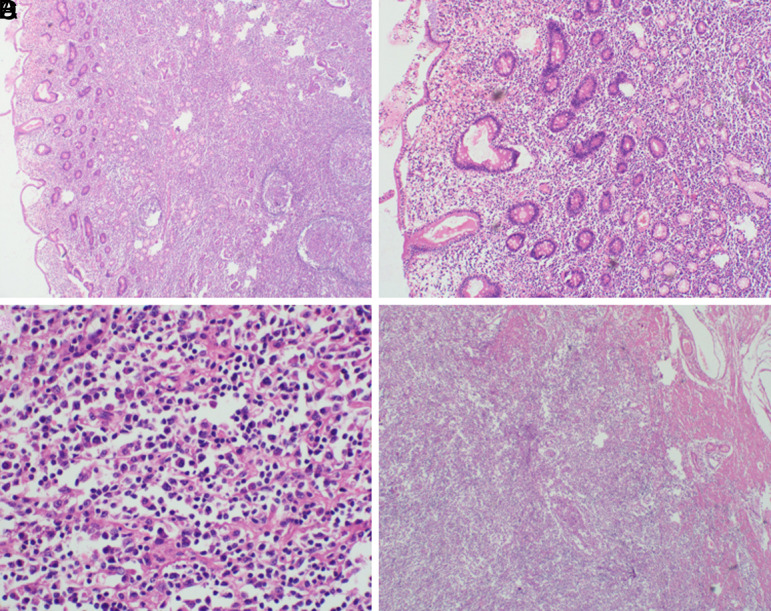
(A) Section from gastrectomy specimen from a case of primary gastric lymphoma showing overlying foveolar epithelium and transmural diffuse infiltration by atypical lymphoid cells, with formation of germinal centers (hematoxylin and eosin [H & E], ×4). (B) Section from gastrectomy specimen showing overlying foveolar epithelium and transmural diffuse infiltration by atypical lymphoid cells. (H & E, ×10). (C) The atypical lymphoid cells are disposed in sheets and are fairly monomorphic, small in size, display round to oval irregular, angulated nuclei, clumped chromatin, inconspicuous nucleoli, and scant cytoplasm (H & E, ×40). (D) The tumor can be seen to infiltrate the muscularis propria (H & E, ×4).

Among 28 patients with PGL, 7 (25%) showed the presence of IM. Histopathological examination revealed gastritis in 17/27 (63%), among whom 14 (82.4%) had antral and 3 (17.6%) had body gastritis. Of these 17 patients, 11 (64.7%) showed mild, 5 (29.4%) moderate, and only 1 (5.9%) showed severe gastritis. Lymphoid follicle was also present among 17/28 (60.7%) patients on histology (Figure 2).

The diagnosis of PGL was confirmed in 15 (50%) patients on endoscopy and in 3 (10%) on cytology obtained by fine-needle aspiration. The diagnosis of PGL was made on histological examination of surgically resected stomach in 12 (40%) patients in all of whom repeated endoscopic biopsies failed to confirm the diagnosis of PGL. Bone marrow core biopsies also showed lymphomatous infiltrate in 2/16 (12.5%) patients only.

Computed tomography data were available in 16 patients with PGL. Using the TNM (tumor [T], nodes [N], and metastases [M]) staging system, 3 patients (18%) presented at stage I, 4 (25%) at stage II with local or distant nodal involvement, and 4 (25%) at stage III with or without invasion of adjacent structures, distant abdominal nodal extension, without distant metastasis. There were also 5 patients (31.3%) who were at stage IV with advanced disease.

Of the 12 patients for whom immunohistochemistry (IHC) data were available, CD20 and leukocyte common antigen were frequently positive among patients with PGL in 9 (75%) and 8 (66.7%), respectively. However, other lymphoma markers like CD3, cyclin D1, cytokeratin, epithelial membrane antigen (EMA), vimentin, B-cell lymphoma 2 (BCL2), CD117, CD34, desmin, and smooth muscle actin (SMA) were positive only in 1 case each. IHC also showed amyloid deposits with kappa light chain positivity in two (16.7%) cases.

*H. pylori *infection was confirmed in 7 (23.3%) and 12 (40%) patients by RUT and histopathology, respectively; 21/29 (72.4%) patients had positive anti-*H. pylori* IgG serology. Using any 2 of the 3 test positive criteria, *H. pylori* was detected in 12 (40%) patients with PGL. Among historical controls with NUD, *H. pylori* infection was detected by RUT in 46/101 (46%), histology in 55/101 (55%), and anti-*H. pylori* IgG serology in 85/101 (84%). The *H. pylori* detection rate was comparable among the PGL patients and historical controls with NUD (*P *= .6).

## Discussion

The present retrospective study suggests that PGL is not uncommon in India. About 40% of the patients with PGL may not be diagnosed even with repeated endoscopic biopsies and require histological examination of surgically resected specimens. *H. pylori* infection was also as common among PGL patients as historical controls with NUD.

*H. pylori* infection is known to have long-term consequences. Chronic *H. pylori* infection has been shown to predispose to gastric atrophy and gastric cancer development. Histological evaluation of gastric biopsies in patients with *H. pylori* infection shows that it significantly predisposes to the development of atrophic gastritis and intestinal metaplasia and subsequently leads to gastric carcinogenesis.^15^ Though gastric adenocarcinoma is the commoner form of GN, PGL is a form of GN with a better prognosis. Hence, its detection has an important clinical consequence.

Compared with a previous study reporting the prevalence of PGL, we found an even higher frequency of PGL, potentially challenging the myth that PGL is uncommon in India.^4^ In our study, almost one-third of the patients presented with non-specific symptoms (dyspepsia with or without weight loss); these results were in line with other studies which have reported epigastric pain as the most common presentation.^15-17^ A family history of cancer was also present in a significant proportion of these patients.

The endoscopic and surgical diagnosis groups were otherwise comparable in their clinical and demographic characteristics, including age, sex, *H. pylori* infection, presentation with dyspepsia with or without weight loss, presentation with organic symptoms, and the need for repeated endoscopic biopsies before conclusive diagnosis was established. To the best of our knowledge, no previous study has compared the endoscopically and surgically diagnosed patients with PGL. Both the groups being compared have major implications as it is not possible to predict based on clinical or imaging characteristics which patients may be diagnosed by endoscopic biopsies or would require surgical resection before the diagnosis can be made (Table 1).

Endoscopic biopsies are superficial, while PGL may involve deeper layers of the stomach; hence, diagnosis may be missed on endoscopic biopsies alone, even after acquiring biopsies using the well technique. Therefore, having a high index of suspicion remains the key to PGL diagnosis. If there is a high suspicion of PGL on endoscopy, resection of the lesion should be done even if repeated biopsies are negative. Multiple studies have previously shown that patients with symptoms of dyspepsia with alarm symptoms like significant weight loss need prompt endoscopic evaluation to rule out malignancy.^18^ The diagnosis of PGL also requires early endoscopy, particularly in those with alarm symptoms.

We did not detect any difference in *H. pylori* positivity among the PGL patients and historical controls with dyspepsia, possibly because of an intimate association of both the conditions with *H. pylori* and *H. pylori* endemicity in this region. Also, lower RUT positivity in the PGL group might be explained by gastric atrophy in patients with malignancy though serology still remained positive.

We diagnosed *H. pylori* infection only if 2 of the 3 tests (RUT, histopathology, and anti-*H. pylori* IgG serology) were positive. In the earlier studies, only histopathology was considered diagnostic of the infection. However, the frequency of *H. pylori* infection in our study was similar to existing literature.^16,17^ Our data show that careful elicitation of clinical history, including alarm symptoms and family history, is important to diagnose PGL. Unintentional weight loss was a predominant symptom at presentation in our study, in contrast with the data from the west, where weight loss is less commonly seen as a presenting symptom.^19^ Multiple studies have shown that endoscopic sensitivity declines substantially for the detection of lymphoma compared to other malignant lesions; our study reiterated that methodically taken biopsies are important for pre-operative diagnosis, and some cases may still require surgery and IHC to establish a confirmatory diagnosis.^19,20^ Immunohistochemistry is required to establish a definitive diagnosis; it has been recommended that all biopsy specimens be subjected to IHC.^21,22^ Although newer diagnostic modalities like texture analysis of positron emission tomography (PET) are coming up, biopsy and IHC remain the gold standard for PGL diagnosis.^23^ Repeated negative endoscopic biopsies may also delay the time to diagnosis and may eventually lead to dismal outcomes.^24^

The inclusion of historical controls, lack of immunohistochemistry data for all the patients, and its retrospective design are some of the limitations of this study.

Primary gastric lymphoma is not an uncommon gastric malignant tumor in India, which contradicts the earlier belief. About half of the patients with PGL may not be diagnosed even with repeated endoscopic biopsies and require histological examination of surgically resected specimens. *H. pylori* infection is as common among PGL patients as historical controls with dyspepsia earlier called non-ulcer dyspepsia.

## Figures and Tables

**Table 1.** Comparison of Demographic and Clinical Characteristics of Non-Surgery and Surgery Groups

**Parameter**	**Non-surgery Group (n = 18)**	**Surgery Group (n = 12)**	***P***
Age in years (mean ± SD)	53.4 ± 13.3	52.7 ± 14.6	.889
Sex	Male: 14 (77.8%) Female: 4 (22.2%)	Male: 8 (66.7%) Female: 4 (33.3%)	.678
*Helicobacter pylori *(2/3 tests positive)	10 (55.6%)	2 (16.7%)	.058
Presentation with dyspepsia with or without weight loss	7 (38.9%)	2 (16.7%)	.391
Need for repeated endoscopic biopsies	2 (12.5%)	4 (33.3%)	.354

SD, standard deviation.
